# From Genetics to Genomics: Facing the Liability Implications in Clinical Care

**DOI:** 10.1177/1073110520916994

**Published:** 2020-04-28

**Authors:** Gary Marchant, Mark Barnes, James P. Evans, Bonnie LeRoy, Susan M. Wolf, for the LawSeq Liability Task Force

**Affiliations:** **Gary Marchant, B.SC., Ph.D., J.D., M.P.P.**, is Regents' Professor, Lincoln Professor of Emerging Technologies, Law & Ethics, and Faculty Director of the Center for Law, Science & Innovation at ASU. He researches, teaches and speaks about governance of a variety of emerging technologies including genomics, biotechnology, neuroscience, nanotechnology and artificial intelligence. Prior to starting at ASU in 1999, he was a partner in the Washington, DC office of Kirkland & Ellis. **Mark Barnes, J.D., LL.M.**, is a partner in the life sciences practice at Ropes & Gray LLP; teaches health care law and the law of biomedical research at Yale Law School; and is founder and co-director of the Multi-Regional Clinical Trials Center (MRCT Center) of Harvard University and Brigham and Women's Hospital. **James P. Evans, M.D., Ph.D.**, is a Medical Geneticist and Internist who is currently retired, but pursued a long-standing interest in genomics and its broad social implications. He is Professor Emeritus, University of North Carolina at Chapel Hill, Department of Genetics. **Bonnie LeRoy, M.S., L.G.C.**, is a licensed genetic counselor with over 20 years of clinical experience. She developed and now directs the Graduate Program in Genetic Counseling at the University of Minnesota. She is a past president of the National Society of Genetic Counselors, the American Board of Genetic Counseling, and the Association of Genetic Counseling Program Directors. **Susan M. Wolf, J.D.**, is McKnight Presidential Professor of Law, Medicine & Public Policy; Faegre Baker Daniels Professor of Law; Professor of Medicine; and Chair of the Consortium on Law and Values in Health, Environment & the Life Sciences at the University of Minnesota. She is a Principal Investigator on the LawSeq project funded by NIH. Institutions are listed for author identification only.

## Abstract

Health care is transitioning from genetics to genomics, in which single-gene testing for diagnosis is being replaced by multi-gene panels, genome-wide sequencing, and other multi-genic tests for disease diagnosis, prediction, prognosis, and treatment. This health care transition is spurring a new set of increased or novel liability risks for health care providers and test laboratories. This article describes this transition in both medical care and liability, and addresses 11 areas of potential increased or novel liability risk, offering recommendations to both health care and legal actors to address and manage those liability risks.

## Introduction

The first two decades of the 21st century witnessed a transition from genetic testing to genomic testing and sequencing in medical practice. Genetic testing (usually of a single gene in a patient) became well established in the second half of the 20th century, leading to enactment of state and federal laws in the United States regulating such testing and the adjudication of litigated cases on issues such as malpractice and negligence in the performance, interpretation, and communication of genetic tests. The late 20th century also saw the emergence of population-wide newborn screening for particular genetic diseases such as phenylketonuria (PKU), with accompanying developments in public health law. However, the completion of a draft sequence of the human genome in 2001 and the emergence and implementation of technologies to perform genomic assessment of patients by testing larger panels of genes, performing microarrays, conducting exome sequencing, and even conducting whole genome sequencing, marked the transition from genetics to genomics in medical care, a transition that is still under way.

The emergence of genomics poses challenges to the established law regarding the practice of clinical genetics. The goal of this paper is to map those challenges in the domain of legal liability and to suggest how state and federal statutes, regulations, and common law should develop to meet the challenges of genomics and to support successful implementation of genomics in clinical care. This paper is part of a larger project funded by the National Institutes of Health (NIH) on “LawSeq: Building a Sound Legal Foundation for Translating Genomics into Clinical Application.”^1^ While this article focuses on the question of how the law of liability should adjust to meet the challenge of genomics, other articles from this project focus on how other aspects of the law should change, specifically the law governing the quality of genomic analysis and interpretation, the law addressing the privacy of genomic data and who has access to results, and the law addressing the boundaries between research, clinical care, public health screening, and direct-to-consumer (DTC) genomics.

This article concentrates on the potential liability of clinicians, laboratories, and health care institutions regarding genomic testing. Other entities in the genomics landscape, such as companies developing and marketing genomic panels and sequencing platforms as well as sequencing instruments and interpretive algorithms also raise important legal questions, including the reliability of those panels and interpretations, FDA status of interpretive algorithms, and contract and commercial liability, but are generally beyond the scope of this article.

This article concentrates on the potential liability of clinicians, laboratories, and health care institutions regarding genomic testing. Other entities in the genomics landscape, such as companies developing and marketing genomic panels and sequencing platforms as well as sequencing instruments and interpretive algorithms also raise important legal questions, including the reliability of those panels and interpretations, FDA status of interpretive algorithms, and contract and commercial liability, but are generally beyond the scope of this article. This article focuses primarily on liability in the context of clinical genomics but also includes translational genomics research that has a clear clinical dimension. Examples of such research are studies involving affected patients, which produce results that are incorporated into those patients' medical records, or used for diagnosis or treatment of those patients.^2^

It is important to note that the law of genetics and genomics builds on and incorporates more general legal principles and precedents. This includes, but is not limited to, state law on liability for medical malpractice and negligence; the law governing informed consent; and principles of vicarious, institutional, and organizational liability that can render health care organizations liable. This article is not arguing for a separate body of law to govern genomics. However, it addresses both state and federal law that include sgenetics-specific statutes and case law involving genetics and genomics,^3^ as well as more generally applicable law. Thus, the paper analyzes new challenges posed by genomics, the adequacy of current law and precedent bearing on liability associated with genomics, and the developments and (in some instances) changes in law required to support successful integration of genomics into clinical care.

The article begins in Part I by elaborating on the current transition from genetics to the genomics. Part II then explores what this transition means for the responsibilities of the various actors in the clinical ecosystem, including clinicians (both generalists and genetic specialists), testing labs, and health care systems and institutions. Part III discusses the role and challenges the liability system will experience in dealing with a rapidly evolving medical field such as clinical genomics. Finally, Part IV describes eleven types of medical liability that are likely to be expanded, modified, or created by clinical genomics. For each of these eleven types of liability claims, we provide specific recommendations for health care actors and institutions and for legal actors and the legal system to manage these liability risks.

### I. The Transition from Genetics to Genomics

The transition under way from genetics to genomics forms the background for analysis of the legal challenges posed by genomics. Genetics focuses on single gene mutations, whereas genomics involves multi-gene tests, usually on a large scale. The frequency of genomic testing has increased in contexts such as cancer risk prediction and treatment, diagnosis of children with puzzling neurodevelopmental conditions, and assessment of what pharmaceuticals should be prescribed for some patients and at what doses. Some clinics and DTC companies are also offering genomic assessment to healthy individuals to assess health risks, including genome-scale sequencing (GSS).^4^ A number of commentators have anticipated the possibility of wider future use of genomics as part of prenatal testing, newborn screening, and general clinical practice.^5^

The emergence of genomic testing has partly been driven by the limitations of genetic testing. Genetic testing primarily consists of testing for variants in specific genes that indicate significant risk for medical conditions such as breast and colon cancer, as well as single-gene Mendelian diseases such as cystic fibrosis, Huntington's disease, sickle cell disease, and Tay-Sachs disease. However, there are relatively few gene variants common in the population that single-handedly confer a major risk of disease. Other than tests for the Mendelian disease genes and a handful of highly-expressed cancer risk genes such as *BRCA1, BRCA2*, and *APC* (adenomatous polyposis coli), few single-gene tests have demonstrated clinical utility.^6^

This limitation largely explains the evolution of genetic science and medicine from single-gene tests to more inclusive genome-wide assays such as gene panels and exome and genome sequencing that assess many or all of a patient's genetic variants. These “genomic” (as distinguished from single-gene “genetic” tests) will sometimes identify a single gene variant that may be clinically significant but may also reveal multiple variants with combined clinical implications, and may aggregate the effect of multiple mutations using an algorithm to provide an overall risk or prognosis score.

The shift from genetic to genomic testing presents new opportunities and complexities. For example, in addition to testing genotypes, genomic testing also enables a broader range of tests, such as mutation assays of tumors or pathogens that are unique to the patient, and gene expression analysis that can help to understand the disease process in a specific patient. Thus, while single-gene genetic testing has primarily been used for risk prediction, disease diagnosis, and assessing carrier status, genomic analysis is enabling additional functions, such as disease prognosis and treatment selection.

The greater applications and complexities of genomic testing relative to genetic testing will undoubtedly impose greater responsibilities on health care providers and institutions. This is likely to lead to new medical malpractice and other liability claims and exposure. The next section explores the expanded responsibilities of health care providers and institutions in the new era of genomic medicine. Then, we briefly summarize the challenges of keeping both medical and legal practice current with the rapidly developing science of genomics. This leads to our analysis of 11 potential legal claims that may eventuate. We provide recommendations on how clinical providers and institutions should respond to these new issues and what legal developments and changes are needed to support successful integration of genomics into clinical care.

### II. New Responsibilities for Health Care Professionals & Institutions

The advent of tools to interrogate the human genome has significant implications for medical practice and will affect a variety of health care professionals, entailing new duties and challenges. Most immediately, those affected will be genetics specialists, such as medical geneticists and genetic counselors, the clinicians already grappling with how best to employ complex new genomic tools including gene panel assays, GSS, and a variety of modalities that are not sequencing-based, such as microarray analysis of gene expression. However, as other medical specialties such as oncology increasingly use genetic and genomic testing in their practices, emerging challenges and responsibilities will fall on a broader array of practitioners, including clinicians who have not been schooled in navigating the complexities of genomic analysis. The demands created by this new class of medical tests will also extend beyond practitioners to those who direct and operate clinical laboratories. Finally, the rise of precision medicine, involving genomic analyses of the general population for purposes of screening or to direct “personalized” treatments for a variety of ailments, will involve still more clinicians, requiring that they be knowledgeable about personalized medicine techniques and their ethical and legal responsibilities in this domain.^7^

A major challenge already confronting those who employ sequencing as a diagnostic test is determining the appropriate breadth of testing to implement. For example, in the realm of hereditary cancer predisposition testing, there are many offerings from commercial laboratories that allow sequencing of a panel of genes, yet the size of those panels varies from 4 or 5 genes to over 80. There is no consensus and no formal policy in either genetics or oncology to guide the choice of panel size so that the scope of testing is appropriate in a given clinical situation. Rather, the onus of that decision falls on the practitioner, who at present is typically the medical oncologist, genetic counselor, or the medical geneticist, perhaps in consultation with the patient's health insurer.^8^ These specialists will be increasingly at risk for accusations of inappropriate or inadequate testing.

Claims of inappropriate scope of testing might not be confined to the scenario in which a practitioner is accused of not testing broadly enough (though this is perhaps the most likely claim). They could also include claims that testing that is too broad and encompasses too many genes, leading to spurious, irrelevant findings that then lead to what might be considered unnecessary “downstream” medical care of a patient and/or family members, such as risk-reducing surgery or surveillance.^9^ This challenge falls most immediately on genetics professionals and medical oncologists, but as testing broadens could conceivably be a threat to generalists as well. Finally, it is possible that the laboratories might be at risk for including genes on offered panels despite insufficient information about those genes and their associated health risks.

The field of genetics previously faced a somewhat similar (albeit more limited) challenge when genetic testing for cystic fibrosis became possible in the 1990s. At that time there was concern that laboratories were offering testing that was too broad and that this would produce an “arms race,” with labs competing based solely on an inappropriate metric of “number of variants” tested.^10^ This could have had adverse consequences for the liability of practitioners, but the threat was largely averted by the formulation of guidelines by the American College of Medical Genetics and Genomics (ACMG), which recommended a “standard panel” of variants to test that was subsequently widely adopted, thus providing needed guidance for the field.^11^

The interpretation of genomic sequencing (and microarray) results represents one of the most significant challenges to the appropriate implementation of genomics in medicine today.^12^ The ability to accurately interpret whether any given genomic variant is actually related to human disease is often difficult (depending on the nature of the disease and the patient's family history) and usually relies on a suite of imperfect measures. These include *in silico* prediction models and functional assays that typically depend upon biochemical knowledge that is still lacking for most genes.

One of the most powerful means by which genetics professionals seek to ascertain the meaning and clinical significance (or lack thereof) of a given variant found in testing a patient is by comparing the variant to data from a variety of existing databases.^13^ If, for example, a variant found in an individual is relatively common in the general population, then it is unlikely that the variant is disease-causing (otherwise a corresponding percentage of the general population would manifest the disease in question). The specific cut-off for what constitutes “too common to be pathogenic” varies for different genes and is the subject of debate.^14^ Further complicating the statistical inference of pathogenicity from population data is the fact that some human genes tolerate variation without adverse impact on function, while other genes are easily disrupted by what would be considered minor variation in another gene.

Finally, the ancestral background of an individual can matter greatly when interpreting a variant using a database. Different populations demonstrate widely variable genomic architecture depending on their ancestry. For example, populations that have been reproductively isolated for many generations (due to either geographical barriers or cultural barriers to reproducing with those outside the group) will have different levels of variation in particular genes. Thus, when interpreting a person's variant using a database, it is crucial that the database includes many individuals from the same ancestral background as that person. All of these considerations make it imperative that large, diverse, and well-annotated databases be created and maintained. Such efforts are under way in the genomics community but remain inadequate at present, complicating the interpretation of genomic variants, especially for those individuals who are of non-European ancestry.^15^ Failure to consult relevant databases when adjudicating variants could clearly be a cause for misinterpretation of variants and thus, legal claims.

The stakes are high when assigning pathogenicity to a variant. When the interpretation is wrong, producing a false positive where the patient is erroneously informed their variant is pathogenic, the patient and family members may be subjected to modalities ranging from surgery to life-long surveillance. Alternatively, where there is a false negative, those who would actually benefit from such interventions fail to receive them. Given the combination of high stakes and limited interpretive capacity at present, there could be liability for both clinicians and laboratorians as a consequence of misinterpretation of variants. The professional genetics community has begun to take action to support quality and mitigate liability by formulating guidelines for variant adjudication.^16^ However, such adjudication remains an error-prone endeavor and one that varies considerably by lab and overtime, virtually guaranteeing that legal cases based on interpretative issues will arise.^17^ This particular liability threat will likely fall on both clinical laboratories and clinicians, and will spread to specialties such as cardiology, ophthalmology, and oncology as those fields embrace such testing.

Given the rapid pace of discovery in genomics, as well as the still highly-imperfect methods available for interpreting genomic variants, there will naturally be some legitimate differences among quality laboratories with regard to variant interpretation. For the time being, the most important indicator of whether appropriate care has occurred will likely be whether the laboratory followed professional guidelines for variant interpretation as promulgated by professional societies such as the ACMG.^18^ Also of relevance will be whether the professionals interpreting variants used appropriate databases^19^ for examining the population prevalence of variants. Even with proper use of such tools, some discrepancy in variant interpretation is to be expected. However, at a minimum, those professionals interpreting variants will be expected to use such resources, or malpractice claims could be legitimately made.

The reinterpretation of the medical significance of genomic variants over time as knowledge advances is an emerging liability threat whose scope has little precedent in other fields of medicine. This threat is especially acute in genomics due to (1) the rapid pace of genomic innovation and implementation, (2) the potential significance to health of genomic findings, and (3) the fact that the genomic sequence itself may remain stable without need to re-sequence, while its interpretation changes significantly.^20^ The field is moving at such a rapid pace that “calling” a given variant as pathogenic or not shifts over time,^21^ and what may have been seen as an innocuous variant two years ago is now considered pathogenic (or vice versa). There exist no clear standards that tell a laboratory or clinician what their duty is, if any, with regard to reexamination of test results generated one, two, or five years ago. Even if genomic results are systematically reexamined at given time intervals, other questions remain with liability implications, such as who will pay for such re-evaluation and who (*e.g.*, the clinician or the laboratory), if anyone, ought to be responsible for re-contacting the patient.

Other changes will be triggered by the widespread clinical uptake of genomics. For example, some envision that pharmacogenomic (PGx) testing will be routinely used in the future to guide the prescription of many drugs with the intent of tailoring individual drug choice and dosage to the individual patient, using their genomic information.^22^ If this vision becomes reality, liability could emerge based upon perceived harm from lack of application, misapplication, or mis-interpretation of PGx information that resulted in the prescription of the wrong agent, administration of the wrong dose, or the failure to prescribe an appropriate and life-saving drug in a given clinical situation.

Another possible legal threat to those implementing genomic medicine derives from choices about what portions of a patient's genetic sequence to analyze. At present some commentators urge sequencing the genome of individuals as a “lifelong resource.”^23^ Given how much of the genome is still not understood, this is often coupled with a proposed strategy to limit analysis and report only on those genes that are well understood. However, once a genome sequence resides in a computer, whether it has been analyzed or not, one can envision a claim that the laboratory and clinician are liable for what they chose **not** to analyze and report. This argument is akin to some objections that have been raised in the debates over reporting of secondary findings.^24^ Such potential legal liability may be an argument for clinicians and laboratories to routinely focus on the analysis of genes that are well understood and to limit GSS to those situations in which casting a broad net is necessary to make a diagnosis. Such a parsimonious approach to testing accords with what has become an axiom of clinical medicine: to seek only information that clinicians truly understand and that will guide their medical actions in the current episode of care.

In addition to the liability of laboratorians and clinicians, health care systems and institutions could be found liable under theories of organizational and institutional liability^25^ if they have failed to provide adequate resources for providers to function in the rapidly changing context of genomic medicine. For example, current electronic health record (EHR) systems are generally poorly equipped to contain, categorize, and display the results of genetic testing, much less genomic testing. As the field moves increasingly to the sequencing of large gene panels and even entire genomes, institutions will need to grapple with how to store such information and how to present it to clinicians and laboratorians who query the medical record of patients in search of genomic information relevant to their patients' care. Moreover, simply storing and presenting such information (as difficult as that may be) is not sufficient. The amount of data in a given genomic analysis is immense and sorting through those data to identify those small pieces that may be of importance to a patient's care is daunting. This reality, coupled with the fact that providers who are not well trained in genomics may increasingly be accessing and using such information, leads to the need for clinical decision support (CDS) systems that proactively alert providers to important information.^26^ For example, when pharmacogenomics information is relevant to prescribing medications for a patient, CDS systems may need to be in place so that an alert is generated in “real time” notifying the provider that potentially critical PGx information needs to be considered. If such systems come into use at major health systems, then other institutions' failure to build and maintain such systems as part of patient safety efforts could ground liability claims.

Finally, many envision the screening of entire populations for genomic risk of preventable disease as part of the future of precision medicine.^27^ For example, there already exist commercial offerings to screen individuals in the general population for *BRCA1/2* mutations^28^ and other high-risk genetic syndromes so as to allow implementation of preventive modalities such as risk-reducing bilateral mastectomy, bilateral salpingo-oophorectomy, or ongoing surveillance. Likewise, efforts are under way to develop expanded panels that would scrutinize genes involved in familial hypercholesterolemia and other conditions in which early treatment could be of benefit. Indeed, there are prominent appeals for GSS of the general population.^29^ Such expansion of genomic analysis into the general population will increase the threat of liability, as applying tests in a low-risk (*i.e.*, general) population will uncover many more false positives than testing affected individuals in the clinical setting.^30^ The legal threats thus increase as genomic testing is applied to the general population.

Recommending how to navigate the liability issues raised by genomics requires considering the intended purposes of medical liability, especially in the context of rapidly evolving medical technology such as genomics. Medical malpractice liability has two intended purposes. First, it is intended to deter health care providers from engaging in substandard and unreasonable health care practices, while incentivizing appropriate uptake of improved medical technologies and practices. Second, medical malpractice liability is intended to compensate those patients who are injured by negligent medical care.

Ultimately, managing the emerging liability threats will require both advances in knowledge (such as the ability to interpret genomic variants properly) and the formulation of evidence-based policies for scope of testing, interpretation of results, and reinterpretation of genomic variants. Yet such advances will take many years. In the meantime, the courts are likely to see a growing number of lawsuits alleging harm from the application, failure to apply, and misapplication of genomic medicine.

### III. The Role of Liability Litigation in an Era of Dynamic Technology Change

Recommending how to navigate the liability issues raised by genomics requires considering the intended purposes of medical liability, especially in the context of rapidly evolving medical technology such as genomics. Medical malpractice liability has two intended purposes. First, it is intended to deter health care providers from engaging in substandard and unreasonable health care practices, while incentivizing appropriate uptake of improved medical technologies and practices. Second, medical malpractice liability is intended to compensate those patients who are injured by negligent medical care.

Notwithstanding general acceptance of these intended purposes of medical malpractice liability, empirical analyses have fueled serious criticism of the medical malpractice system in practice. The medical malpractice system fails to compensate the majority of patients injured by negligent health care.^31^ Moreover, the compensation paid in damages is poorly correlated with the harm caused by malpractice.^32^ The threat of liability, especially when the expectations of medical malpractice doctrine are unclear, may lead providers to protect themselves from liability by engaging in defensive medicine, which can subject patients to unnecessary procedures and treatments.^33^

Medical malpractice liability is particularly challenged by fast-moving technologies, such as genomics. Empirical studies have demonstrated that new technologies are one of the most powerful drivers of malpractice liability.^34^ This is because physicians and other providers are often unfamiliar with the new technologies and thus make more frequent mistakes, plus there is differential uptake of the technologies, which can lead to disputes over whether the standard of care at a given time requires use of the new technology.^35^

When a technology is evolving as fast as genomics, providers' skill set and knowledge base can be quickly outdated, resulting in clinicians practicing with inadequate capabilities. This is already happening with genetics and now genomics — most physicians practicing today received little genetics training in medical school, and so are being left behind as genetics and genomics assume increasing importance in health care delivery.^36^ Physicians may be shielded from liability by the traditional custom-based standard of care, in which physicians are held to be complying with the standard of care if they are acting consistently with what other similar providers (such as other physicians in the same specialty) would do. This protection will be most effective in jurisdictions applying a local, rather than a national, standard of care.^37^ However, this liability life jacket is gradually deflating, as an increasing number of states are moving to a more objective, reasonableness-based standard of care rather than one based on custom, and a growing number are embracing a national standard of care.^38^

The clinical and knowledge support systems that clinicians use to stay current may lag in a time of rapid technology change. For example, professional societies and others promulgating clinical guidelines, which provide guidance on best medical practice in many different fields of medicine, may struggle to stay current with new genomic science. These guidelines can take months if not years to be developed and approved, but may be obsolete by the time they are published because of the dynamic changes in genomic science. The inability of clinical guidelines to keep pace with rapidly evolving genomic science puts clinicians in jeopardy. The problem is exacerbated when other practice aids such as CDS systems also fail to keep pace. Yet, when dealing with such a rapidly evolving area of clinical medicine that is new to many practitioners, up-to-date guidelines will be critical to support sound practice and to help the legal system evaluate clinical decisions and actions.^39^

One step toward greater clarity on expectations would be to see jurisdictions that still use a local standard of care move toward a national standard.^40^ Good clinical practice related to genomics should be consistent across the country, and so the standard of care should not vary from state to state. In addition, patients may get their care in multiple states, even in a single episode of care.

Genomics is provoking not only changes in clinician practice, but also changes in what is expected of health care institutions. Modern health care, driven by big data and genomics, is becoming much more of a systems-based practice. A patient's care may involve several providers, including the primary care physician, one or more specialists, and perhaps a genetic counselor. Laboratories that analyze and communicate genetic and genomic results from a patient's samples play an increasingly central role in modern health care. Finally, health care institutions must provide the necessary equipment, data sources, and practice aids to support modern molecular health care. Along with these greater responsibilities for labs and institutions come new liability risks. It is not only the knowledge and practice of the individual physician that is changing, but also the evolution and coordination of an entire health care ecosystem around each patient, creating new liability risks.

In the next section, we identify 11 key claims that lawyers may consider bringing as patients experience the transition to genomic medicine. These claims raise overlapping issues, but parsing those claims helps identify the liability challenges raised by new and expanded responsibilities for health care providers, laboratories, and health care institutions. After analyzing changing circumstances and responsibilities, we recommend how health care actors and institutions should cope with these responsibilities to control their liability exposure. We then address what legal development and changes are needed to support successful implementation of genomic medicine in clinical care. As noted in the introduction, in some cases the legal issues are comparable with those presented in other types of diagnostic malpractice cases, with perhaps just minor perturbations needed to address the unique aspects of genomics, whereas in other cases the legal issues are more novel. In addressing all these genomic liability issues, we strive to balance the important role of liability in encouraging uptake of new and better technologies to incentivize good care for patients, against the potential downside of liability in discouraging innovation and pushing providers to undertake defensive medicine and order unnecessary tests that risk imposing unnecessary costs and detracting from patient well-being.

### IV. Potential Liability Claims

This part analyzes 11 potential claims of liability that may be advanced as genomics is integrated into clinical care. **Table 1** shows the roster of claims analyzed below. Though these claims may raise overlapping issues, we analyze them individually, as attorneys bringing suit may distinguish among them in deciding what specific legal claims to bring. Yet, it is worth noting that a pervasive source of uncertainty and potential liability for health care providers and institutions is the need for better evidence and guidelines to support sound practice in this time of evolving genomic science.^41^ As elaborated in other scholarship from the LawSeq _proj_ect, there is a need for provisional advice to clinicians (both physicians and general counselors)and laboratories that recognizes the uncertainties in determining risks and benefits of genomic testing.

**Table 1.** Potential liability claims with the integration of genomics into clinical care.

1	Failure to Test
2	Over-Testing and Incomplete Information
3	Choice of Specific Panels or Tests
4	Inappropriate Use of or Reliance on a Test
5	Incorrect Variant Calls
6	Failure to Communicate Results to Patients Accurately
7	Failure to Communicate Results and Share Data with Clinicians within a Heath Care System
8	Failure to Analyze and Offer Incidental Findings or Secondary Results
9	Failure to Update and Recontact
10	Failure to Warn Family Members
11	Error and Failures in Direct-to-Consumer (DTC) Testing

This provisional advice would be subject to regular updating in response to evolving evidence and would have several components. First, it would address credentialing, by addressing who within health care systems should be able to order different types of genomic tests. Second, there should be guidelines, perhaps more transient and less developed than traditional clinical guidelines, which are developed cooperatively by all relevant stakeholders, providing provisional recommendations on genomic testing at a time of incomplete and uncertain evidence. Finally, there should be opportunities and encouragement for health care systems and laboratories to contribute to evidence generation. These goals and principles help guide the recommendations provided for the 11 specific legal issues discussed below.

#### 1. Failure to Test

##### A. analysis

Failure to test in the genomics era will likely mirror what we already see with genetic testing in medicine. Mostly, failure to test will be the result of the provider's failure to recognize a risk in an individual. In many cases, family history is the known indicator that there may be an inherited risk for specific individuals, even if this family history does not follow a Mendelian pattern. However, in the case of many conditions (most of them rare) finding the gene or genes involved is difficult and in the absence of a clear family history through multiple generations, risk to individuals is often missed. Of course, such a failure will result in liability only if it fails to meet the standard of care — not all adverse outcomes or wrong choices are the result of negligence.

A classic example of failure to test involves the belief on the part of the patient, and often the clinician, that pathogenic variants in breast cancer genes cannot be passed down through a male in the family. A clinician may also fail to consider genomic testing for an individual who is the only person affected in a family where everyone else is healthy. It is not uncommon for clinicians to fail to consider that a single affected person might have a genetic condition and therefore fail to test that patient. This could result in failure to make a diagnosis for the affected individual and failure to identify others in the family (including potentially future children) who are also at risk.

There is a lack of guidelines for when to use genomic testing and how best to interpret and manage results. Genomic testing is not always the best choice, interpreting the results is often confusing, and the diagnostic yield is variable depending upon the indications for testing and laboratory that completed the testing. How to follow up on a test that failed to yield a diagnosis may not be clear, and the fact that the results may look different when coming from different laboratories complicates the decision to test. Lastly, the literature continues to show that health care providers not trained in genetics lack the knowledge and skills needed to assess risk.^42^

##### B. recommendations

Recommendations for Health Care Actors or Institutions:

- In order to ensure quality care and to prevent liability, health care organizations should determine who within their organization is qualified to order genomic testing. Appropriate training is necessary to understand the type of genomic test to order (*e.g.*, gene panel vs. GSS), communicate the results to patients, and incorporate the results into patient care. If a clinician does not have relevant training, the clinician should refer the patient or obtain a clinical consult with a provider (physician or genetic counselor) who does have the requisite training.
- Professional societies should give higher priority to issuing clinical practice guidelines, guidance, and/or recommendations to assist clinicians in making genomic testing decisions. In the absence of updated clinical guidelines, health care organizations and professional societies should provide guidance on appropriate use of genomic testing that incorporates the perspective of all relevant stakeholders.
- Health care institutions have responsibilities to offer or facilitate access to (1) continuing education on these topics, (2) genetic and genomic laboratory capacity and professionals, and (3) clinical processes (including clinical decision support (CDS) and adequate electronic health record (EHR) representation of genetic and genomic tests results) to support good clinical practice.
- Genetic counselors have a crucial role to play in clinician, patient, and family education and counseling.
- When genomic testing is indicated, the clinician should discuss this with the patient and offer a referral if the patient's health care institution does not offer genomic tests.

Recommendations for Legal Development and Change:

- When clinicians are accused of negligence or malpractice for failure to order genomic testing, attorneys and courts should access and consider clinical practice guidelines (CPGs), other recommendations and resources from professional societies, and the relevant medical literature, which may be germane, to but not determinative of, the standard of care.
- Because genomics is a developing field, doctrines recognizing multiple schools of professional thought may be relevant to adjudication. Highly competent genomics professionals may disagree on the question of when testing is indicated, the appropriate type of testing, and the interpretation of results.
- In some domains of genomics, standards are already clear on when testing is indicated. When that is not the case, it would be helpful to have professional guidance stating current uncertainties and options in order to make clear that providers must make professional judgments in light of uncertain evidence. In such situations of uncertainty, health care institutions and providers can protect themselves from liability by documenting that they had in place a reasonable process for deciding whether and what kind of genomic testing is warranted in light of the uncertain evidence, and that they explained the available choices to the patient.

#### 2. Over-Testing and Incomplete Information

##### A. analysis

When considering testing, it often seems that more is better. The more data that can be gleaned from genomic testing, the greater the likelihood of an answer for the patient and the family, or so one would think. However, in the current state of genomic testing, this is often not the case. Whole genome and exome testing can lead to information that is not wanted, does not aid the diagnosis or management of the case, and cannot be interpreted. In some cases, the results of GSS can give results that can be misinterpreted or over-interpreted, which can potentially harm the patient by encouraging additional testing and even medical procedures that may not be warranted and might even be dangerous.^43^

An exome as opposed to a targeted test does not have the same coverage of potentially critical genomic regions. Exome testing could miss the critical area that might lead to a diagnosis. Exome testing sequences the coding regions of the genome, covering about one to two percent of the genome.^44^ It is designed to provide a certain depth of coverage to ensure accuracy.^45^ However, this is less than the coverage that would occur when testing for a specific gene. The increased potential for error in exome sequencing could result in failure to identify an area critical to diagnosis. In contrast, a targeted panel is more comparable to a single gene test. It is more specific, and the coverage is higher. A significant risk to clinicians is believing that whole genome or exome sequencing “looked at everything” and thus assuming that if nothing was found, then there is no genetic basis for the phenotype. This assumption may be incorrect.

##### B. recommendations

Recommendations for Health Care Actors or Institutions:

- Clinicians should determine the scope of testing to order in a particular case by considering clinical practice guidelines (if any), other recommendations from professional societies and the scientific literature, and consultation with their testing laboratory.
- Clinicians ordering genomic analyses should be familiar with the testing options, their characteristics, and the trade-offs among them, in order to make a well-grounded determination of which tests to order.
- The scope of testing or sequencing ordered should optimize clinical utility (*e.g.*, diagnostic yield), while minimizing results that are not relevant to the indication for testing. Parsimony in testing can be beneficial to prevent false positives and results that may be misinterpreted or over-interpreted.
- If clinicians, their laboratory, and/or their health care institution consider enlarging the scope of testing to include a set of secondary or incidental findings (*e.g.*, those recommended by ACMG^46^), this should be discussed with the patient as part of the informed consent process before testing. The patient should have the option of refusing some or all of these added analyses.

Recommendations for Legal Development and Change:

- Courts should recognize the over-testing (*i.e.*, ordering tests not called for by the patient's symptoms, conditions, or history) can produce false positive results that can harm patients by requiring additional follow-up procedures that may prove iatrogenic.
- Most genetic malpractice cases to date involve a provider's failure to test for a particular genetic condition or variant. However, due to the uncertain nature and large volume of data generated by genomic tests such as GSS, it may be negligent to recommend over-testing in some situations.

#### 3. Choice of Specific Panels or Tests

##### A. analysis

Genomics creates an additional level of complexity compared to genetics in requiring that a physician or other health care provider choose which specific test(s) to order for a particular patient. In the past, the provider would assess whether the patient had a predisposition or illness that might be informed by genetic testing, and then would recommend a genetic test for that condition. Typically, this would involve testing a single gene. For example, a physician or genetic counselor may recommend carrier testing of a prospective parent for a mutation in the *CFTR* gene associated with cystic fibrosis or testing for mutations in the *APC* gene for a member of a family with a history of familial adenomatous polyposis (FAP). While different test manufacturers or labs may offer their own test for that particular gene, perhaps with different variants assayed by the test, there is usually no issue as to what gene to test for, only which test might include the most robust and relevant set of variants for that single gene.

An important difference in the genomic era is the shift from testing of a single gene to testing panels of genes or even exome or genome sequencing.^47^ With the realization that the risk of almost all diseases (at least all non-Mendelian diseases) is affected by many genes, a patient believed to be at risk for a specific disease can best be assessed with a gene panel that packages into one test platform assays for a number of different genes that may affect the risk of that particular disease. Disease risk may be determined by “hits” within specific genes included in the panel, or by an algorithmic score that takes into account the testing results for all the genes in the panel. Test manufacturers and testing laboratories are increasingly using gene panels to test patients for a specific disease risk such as GeneDx's panel for epilepsy, which currently includes over 200 genes associated with multiple disorders that may involve they symptoms of epilepsy. As more genes are found to be associated with epilepsy, companies can add those genes to the panels. Another example is the CustomNext-Cardio panel by Ambry Genetics.^48^ In this case, the panel allows the clinician to choose a group of disorders from a list of potential diagnoses such as lipid disorders, aortic aneurysms, cardiomyopathies, etc., to test for pathogenic variants associated with these diagnoses.

The novel liability risk associated with gene panels is that the health care provider may face liability if he or she recommends an inferior panel for an individual patient, and it turns out that the testing misses an important variant that would have been detected if a better panel had been recommended. Bringing a lawsuit for such a faulty recommendation would present some practical proof problems. The plaintiff (or their attorney) would need to discover that another gene panel was available and would be covered by the patient's insurer or funded by the patient, and that the alternative panel had genes in it that would have been more informative and affected the outcome if the physician had recommended that panel instead. Often potential plaintiffs and their attorneys will not be aware of the potential consequences of testing with a different gene panel, but if this is discovered by a plaintiff's expert, for example, who finds that the plaintiff was adversely affected by the failure to detect a mutation that was included in an alternative panel, this could be the basis of a potential medical malpractice claim. Specifically, the plaintiff would need to show that (1) an alternative gene panel was available and would have been utilized by the patient, (2) that this alternative panel would have disclosed information that would have resulted in a better outcome for the plaintiff, and (3) the physician should have had some reason to suspect that the alternative gene panel would have produced a superior result. This opens an additional potential claim for a plaintiff alleging genomic malpractice.

The choice of an appropriate panel may also implicate the lab or institution. Traditionally, the clinician orders a test (often designated by the patient's health insurer), and the lab performs that test and reports results back to the physician. As we move into the genomic era, however, the relationship between the clinician and test lab is becoming more complex and variable. In some cases, the lab may provide feedback or recommendations on the test panel the physician ordered, sometimes suggesting a different panel or perhaps adding in a few additional genes based on the individual patient. As labs perform this advisory role, physicians may actively consult the lab for recommendations on what gene panel to order. The choice of gene panel will be influenced by a number of factors in addition to the genes present on each panel, including each panel's coverage of relevant gene variants, the rate at which they detect specific variants, their cost and available insurance coverage, and how good they are at interpreting variants of uncertain significance (VUSs). In other institutions, the labs play an even more active role, and are in active partnership with clinicians in the patients' care. In still other contexts, the lab has developed its own test panel, and gives the physician the binary choice of whether to order that test panel or not. Depending on how active a role the lab had in selecting the specific test panel, the lab may assume some or all liability for an erroneous choice of a test panel. The health care institution may also bear responsibility if it creates a process or committee that reviews or recommends test orders.

##### B. recommendations

Recommendations for Health Care Actors or Institutions:

- Given the uncertainty and rapid evolution of genomic tests, greater transparency on the evidentiary basis and performance of available genomic tests would be highly beneficial, including those commercially available.
- The government or professional societies should publish and regularly update a listing and comparison of test panels and sequencing approaches that can be used to evaluate a particular genetic risk.
- Professional societies should whenever feasible identify specific tests or test parameters that are recommended for patients with specific risk profiles, and physician compliance with those recommendations should be evidence of reasonable care. These recommendations need to be updated on a regular basis as new tests and evidence become available.
- When the gene variants or algorithms incorporated in a test are changed, there should be a process and standardized format to notify providers of this change and the basis for the change.

Recommendations for Legal Development and Change:

- Given the trade-offs that may be inherent in constructing genetic test panels and algorithms, there should not be liability for test selection simply because it produces an inferior result in a specific patient.
- The choice of a test is particularly prone to the well-known phenomenon of “hindsight bias” in litigation,^49^ in that various testing options may have strengths and weaknesses that may look very different before and after the testing is done and outcomes occur. Courts should restrict, under the rule against prejudicial evidence, attorneys from insisting that all negative outcomes show malpractice and should instruct juries carefully to avoid hindsight bias.
- The learned intermediary doctrine^50^ should be interpreted as requiring test developers to be transparent with ordering clinicians and labs about the strengths and weaknesses of their tests in order to get protection from liability under the doctrine.

#### 4. Inappropriate Use of or Reliance on a Test

##### A. analysis

The shift to genomic medicine will provide a much larger set of data on which health care providers can rely for disease prediction, diagnosis, prognosis, and treatment. The availability of data from genomic tests will inevitably lead to second-guessing of how a provider used that data in a patient's care, especially when the patient had a bad outcome.

An example is reliance on a genomic test that predicts recurrence of breast cancer, and thus whether a patient should undergo chemotherapy after surgical removal of a tumor. In the past, many breast cancer patients unnecessarily incurred the costs and side effects of chemotherapy, because no information was available on their individual recurrence risk. Now genomic tests such as Oncotype Dx and MammaPrint predict the recurrence risk for an individual breast cancer patient. These tests are probabilistic not deterministic, and thus cannot predict with certainty whether a breast cancer will recur, but rather provide a probability known as a recurrence score. If a treating physician recommends that a patient undertake such testing, and if (for example) the test provides a low recurrence score so that the patient on the advice of the physician forgoes chemotherapy, can the patient sue the physician if the cancer nonetheless recurs? Or alternatively, if a physician fails to recommend such a test, and the test would have indicated a high recurrence score that would have indicated chemotherapy, would a patient have a claim against the physician for failing to recommend such a test?

An important principle of medical malpractice is that a physician is not, and should not be treated as, a guarantor of the patient's good outcome.^51^ Most medical procedures and decisions involve inherent uncertainties and risks, and sometimes the treatment will result in a bad outcome even if the physician's judgment to recommend that course of treatment was reasonable at the time the decision was made. Nevertheless, when a patient does have a bad outcome, there will often be a temptation to second-guess the physician's decision on whether or not to have recommended a genomic test that may have resulted in a different outcome.^52^ A practical difficulty in bringing this type of lawsuit is that a patient may never know if their result would have been different if the test had or had not been recommended, because the test results are probabilistic rather than deterministic.

At least one such case has already been reported. In that case, a breast cancer patient was diagnosed as having a non-invasive cancer, which was treated but later returned and metastasized. The patient alleged that if she had been given the Oncotype Dx assay her tumor would have produced a recurrence score of 41, which would have indicated the need for chemo-therapy, which may have prevented the tumor recurrence.^53^ She brought a medical malpractice lawsuit against her physician for failing to recommend an Oncotype Dx assay, and the case eventually settled.^54^

##### B. recommendations

Recommendations for Health Care Actors or Institutions:

- The lack of clinical data to evaluate predictive value and clinical utility for many genomic tests presents a challenge to providers in determining whether a particular test is valid and reliable. Researchers, test developers, regulators, payers, and professional societies should work to ensure that sufficient clinical evidence supports available genomic tests, including those commercially available.
- Professional societies and others generating clinical practice guidance need to provide physicians with clear recommendations regarding what genomic tests are ready for application in the clinic, and for what indications or risk profiles.
- The reliability of genomic tests will depend on the robustness of the data on which they are based, including data that are representative of the patients for whom the test is recommended. Test developers and research funders should ensure that genomic tests are based on data from a diverse and representative cross-section of the population.
- Physicians need to explain carefully the limitations and uncertainties associated with the specific genomic tests they recommend to their patients, so the patient can make an informed decision on whether to assume the risk and make medical decisions on tests that are inherently imperfect (at least for the time being).

Recommendations for Legal Development and Change:

- Many genomic tests will give probabilistic rather than deterministic outcomes — in other words, the test will indicate that patients may have a certain risk. The fact that an individual patient has a bad outcome is not in and of itself evidence of negligence, and in this area of clinical practice as in others, the clinician cannot be a guarantor of the patient's positive outcome. Rather, the negligence inquiry should focus on whether the clinician's recommendation of and reliance on a test was reasonable in the circumstances of the individual patient and consistent with the prevailing standard of care.
- A key factor in whether a provider is negligent in recommending or not recommending a genomic test for a particular patient is whether the provider adequately explained the options, uncertainties, and risks of different courses of action. Documenting such discussions will often be critical to defending against liability.

#### 5. Incorrect Variant Calls

##### A. analysis

Determining whether a particular gene variant is of clinical significance, and what should be communicated to the patient, is a major challenge in genomic medicine, particularly when exome or genome sequencing is conducted. Such sequencing will invariably (at least for the foreseeable future) generate a large number of VUSs.^55^ Both the testing laboratory (which usually makes the initial “call” on the clinical significance of variants) and the patient's physician or genetic counselor who communicates the test results to the patient will have responsibilities that may be second-guessed in litigation.

There is often inconsistency between reference databases on whether a particular variant is pathogenic, likely pathogenic, or of uncertain significance. If a test laboratory or provider reports to the patient that a variant was of uncertain significance (a VUS), when in fact the variant turns out to be clinically significant and the delay in understanding the risk results in injury, the patient could bring a lawsuit claiming the lab or provider erred in classifying the variant as a VUS. The playing field in such cases may be tilted against the defendant if subsequent information makes clear that the variant is significant — things always look clearer in hindsight, even though the legal fact-finder is supposed to evaluate the decision based on the information available to the defendant at the time the challenged action occurred. If the lab or provider did not report the variant to the patient at all because it was deemed to be of uncertain significance, this action could again be second-guessed in a legal claim for failure to disclose.

The first case presenting such issues is currently pending in South Carolina federal court—the *Williams v. Quest* case.^56^ In that case, a child suffering from seizures was genetically tested, and a variant determined to be of uncertain significance was detected in a potentially relevant gene known to be associated with epilepsy (*SCN1A*). The child died from his condition in 2008, after being treated with a drug that is now known to be contra-indicated by the gene variant carried by the child. The testing lab issued a revised report in 2015 re-categorizing the VUS as a pathogenic mutation. The mother subsequently brought a lawsuit contending that the testing lab should have known the variant was pathogenic at the time it issued its original report, or that it should have updated its report promptly when it did discover that the variant was pathogenic and communicated that revised result to the patient.

##### B. recommendations

Recommendations for Health Care Actors or Institutions:

- Inconsistent classifications of the same variant by different sources and databases present a major problem for communicating test results to patients. Ongoing efforts to reconcile or integrate different variant databases will be very helpful in providing clearer guidance to labs and physicians in interpreting the clinical significance of genetic variants.
- A physician should be responsible for communicating and explaining to a patient any variants reasonably classified as pathogenic or likely pathogenic that are reported to the physician by the lab in response to the physician's order for genomic testing.
- The physician should also be able to explain “negative” results (benign or likely benign) if relevant to the patient's condition, risks, or concerns.
- There is controversy over whether and when providers should communicate VUSs. When the patient has a family history or other evidence of a specific genetic problem, and testing of the relevant gene(s) indicates only VUSs, the physician should communicate information about the specific VUS if clinically appropriate and urge the patient to continue to follow-up on the relevant variant(s) going forward in future interactions with health care providers.
- Policy makers, health care institutions, and professional societies should seek to integrate different interpretive databases to provide the most robust and consistent evidence on the pathogenicity of genetic variants.

Recommendations for Legal Development and Change:

- Courts and attorneys should pay careful attention to clinical practice guidelines and other recommendations and guidance from professional associations on making variant calls. These resources provide helpful evidence on reasonable practices even if they do not conclusively establish the standard of care.
- A physician may be held liable for failing to disclose and explain a variant that has been identified as “pathogenic” by the test laboratory. However, if the lab uses more than one database for making variant calls, and reports that the databases give inconsistent results on whether a particular variant is pathogenic, the physician generally should disclose to the patient both the pathogenic and non-pathogenic determinations and explain why different databases may give inconsistent results and what this means for the patient. A physician may offer their own professional opinion as to why a particular database result may or may not be compelling.
- A physician should not have a legal duty to go beyond the lab report and look at other sources that may classify a variant differently, unless that physician has good reason to know or suspect there are different interpretations available. Failure to disclose alternative variant interpretations should be the responsibility of the lab, not the physician. As with its other testing operations, a laboratory should have a legal duty to consult known and respected data sources in providing variant interpretation to the physician (and ultimately to the patient).
- A physician should not have a general duty to communicate and explain specific VUSs to a patient. However, in cases where the patient is known to be at risk of a disease, the physician should explain VUSs in genes of relevance, caution that some of these variants may become of known significance in the future, and inform a patient that he or she should consider seeking re-interpretation of these test results at a later time.

Genomics will dramatically increase the challenges in communicating information to patients. Even in past genetic testing examining a single gene, many patients had difficulty understanding the results, as those often involved probabilistic and sometimes uncertain risk estimates. Communicating genomic information that may involve dozens or hundreds of gene variants will be even more confusing for patients.

#### 6. Failure To Communicate Results To Patients Accurately

##### A. analysis

Genomics will dramatically increase the challenges in communicating information to patients. Even in past genetic testing examining a single gene, many patients had difficulty understanding the results, as those often involved probabilistic and sometimes uncertain risk estimates. Communicating genomic information that may involve dozens or hundreds of gene variants will be even more confusing for patients.

In addition, physicians who are not specialists in genomics will experience increased difficulties in comprehending genomic information. Again, studies have shown that physicians often do not understand genetic information involving just one or two genes.^57^ Now with the even more complex data from genomics, non-specialist physicians and other providers will be hard-pressed to understand the data and its significance, much less to communicate that information to patients in an accurate and understandable manner. This creates a risk that the clinician will misinterpret and incorrectly communicate the test results and their significance to the patient. Even genetic counselors and physicians with expertise in genetics may have trouble understanding and communicating more complex genomic tests if they do not obtain additional training.

For example, consider a physician seeking to report the results of an exome sequence back to a patient. There may be thousands of variants in any patient's exome that differ from the reference sequence, though a relatively small percentage will be associated with known genetic traits. Of those, an even smaller percentage will be of medical significance to that patient and recognized as “pathogenic.” But a “pathogenic” classification may be tenuous and involve uncertainties that the provider will need to communicate to the patient.^58^ In addition, different databases may classify the same variant differently as to its pathogenicity, and those classifications may change with time as new data are collected.

Given this complexity, along with the limitations of providers' and patients' understanding, conveying this information from provider to patient in an accurate and understandable manner will be a major challenge for the foreseeable future. If the patient fails to grasp the full significance of what is conveyed, and based on that misunderstanding makes treatment, reproductive, or behavioral choices that result in adverse outcomes, the patient may seek to sue the provider for inadequate communication of risks or information. In the genetic era, such lawsuits for failure to adequately communicate or disclose genetic information were common.^59^ This type of misunderstanding and subsequent lawsuit is likely to be even more frequent in the genomic era. In genetic malpractice cases, the physician's medical chart notes of what was communicated to the patient verbally could be critical for resolving such disputes — that is likely to be even more the case in the genomic era.

##### B. recommendations

Recommendations for Health Care Actors or Institutions:

- Clinicians are responsible for communicating to patients the results of genomic tests that the clinicians have ordered, and doing so accurately. Accurate communication requires an adequate understanding of genetics and genomics and the specific tests results.
- Clinicians may need assistance from genetic counselors and medical geneticists to explain the need for and results of genomic testing.
- Information about the nature and meaning of genomic results, and about how to communicate them accurately and meaningfully to patients, should be integrated into medical school curricula and post-graduate training programs. Providers should consider delivering genomic results to patients both orally and in writing to aid comprehension and allow patients to seek further assistance in understanding their results.
- Laboratory reports should be in a form that is accurate and can be understood by the average clinician without specialty training in genetics and genomics.

Recommendations for Legal Development and Change:

- Many medical malpractice cases against physicians for alleged negligence relating to genetic testing involve factual disputes about what the provider told or did not tell the patient. Courts should expect providers to document in patient records the information that was communicated to the patient about the need for and results of genomic testing.
- Providers have a legal duty to communicate clinically relevant genomic information to their patients in a manner that is both understandable and informative.

#### 7. Failure to Communicate Results and Share Data with Clinicians within a Health Care System

##### A. analysis

The increased complexity of genomics relative to genetics will increase the responsibilities of health care providers to communicate and share relevant genomic data and related practice resources within their systems. For example, over time the case for pharmacogenomic (PGx) testing to guide drug prescribing will likely become stronger,^60^ particularly for patients who have already had genetic sequencing, allowing their providers to use that data to identify relevant variants affecting drug metabolism.^61^ However, for this to work, the health care system must ensure that two types of information are easily accessible to providers in their system. First, the relevant variants affecting drug metabolism in a patient's sequencing data must be flagged by the lab or clinical software so that the prescriber will be notified of those variants in the patient's genome. Second, the PGx variants that are known to have clinical utility must be listed and linked to the individual patient's profile in the EHR when a prescription is written.

Some health care systems have begun developing CDS systems to help identify and flag relevant PGx variants for providers.^62^ However, most others have not, especially at smaller and medium-sized systems. If a patient has a bad reaction to a drug (or a failure of drug effectiveness) that could have been prevented by PGx-based prescribing, by either prescribing a different dose or a different drug to a genetically at-risk patient, there may be a malpractice lawsuit.^63^ This lawsuit could target the provider for failing to utilize the relevant genomic information in prescribing the drug, but could also target the health care system for failure to put in place a reasonable CDS system that would ensure the most relevant information is flagged for the prescribing physician.

However, both medical and legal decision-making in the field of PGx is complicated by the slow uptake of such genomic testing in clinical practice.^64^ This slow adoption of PGx testing can be attributed to a number of factors, including lack of proven clinical utility in many instances, physician unfamiliarity and resistance to such testing, and the lack of payer coverage.^65^ The FDA has now put genomic information on the “labels” (patient package inserts) of well over 100 different drugs.^66^ However, an FDA label does not determine the standard of care, although it may provide relevant evidence, and many if not most physicians are not (yet) following the genetic testing guidance on many drug labels.^67^

There are a handful of cases where it would likely be malpractice today if a physician prescribed a drug without a genetic test and the patient had a bad outcome. Examples include HLA-B testing for the anti-HIV drug abacavir,^68^ and testing for *TPMT* genetic polymorphisms before treating childhood leukemia patients.^69^ However, other notable examples such as clopidogrel and warfarin, once thought to be the poster children for PGx testing, and which have FDA recommendations for genetic testing on their labels, are not routinely being used with PGx testing in clinical practice today.^70^ Part of the problem is that the delay and cost (unreimbursed) of PGx testing may hamper its use, but this situation may change if patients have already been tested for the relevant PGx variants in DTC testing or with genome sequencing, potentially allowing the clinician to check the patient's genetic susceptibility before prescribing the drugs with no additional costs or delays. In addition, the rise of GSS is likely to accelerate the potential for PGx testing by enabling the detection of a more comprehensive set of relevant PGx markers.^71^

##### B. recommendations

Recommendations for Health Care Actors or Institutions:

- Optimally, health care providers should have access to clinical decision support (CDS) systems that indicate genetic variants (and genetic or genomic tests where appropriate) that should be considered when providers within their system prescribe treatments.
- A health care system that uses sequencing data in clinical care should seek to develop CDS and electronic health records (EHR) systems for ensuring that a sequenced patient's clinically significant genetic variants are made available to physicians in an easily accessible format.
- As the field of health informatics and genomic testing changes rapidly, institutions should track emerging trends in information systems and legal expectations.

Recommendations for Legal Development and Change:

- FDA warning labels, clinical guidelines, and other professional society guidance should provide probative but not conclusive evidence of standard of care for pharmacogenomic (PGx) testing. Professional societies should seek to produce regularly updated guidance that incorporates the most recent information to help guide decisions by clinicians and laboratories.
- In determining the evidentiary case for requiring PGx testing, courts should give the highest weight to peer-reviewed, published, randomized control trials that demonstrate clinical utility. Peer-reviewed observational studies and meta-analyses can also be considered but should be given less weight. Anecdotal reports and non-peer reviewed studies should not factor into the evidentiary weighing.
- A stronger case for institutional or professional liability is present when a patient has previously been genetically tested or sequenced for the relevant polymorphisms affecting drug susceptibility, and the results are readily available to the health care provider, but the provider does not take this available information into account in a prescribing decision that results in harm to the patient.

#### 8. Failure to Analyze and Offer Incidental Findings or Secondary Results

##### A. analysis

Incidental or secondary findings (hereinafter called “incidental findings”) in clinical genomics are “genomic variants of potential medical relevance unrelated to the medical reason for ordering the test.”^72^ It is widely recognized that genomic sequencing will routinely generate incidental findings, depending on the scope of that sequencing and subsequent analysis.^73^ A robust literature now addresses return of genomic results and incidental or secondary findings in a range of contexts — research,^74^ clinical care,^75^ and public health screening.^76^ We focus here on management of incidental findings in clinical care, addressing findings initially identified in clinical sequencing or public health screening (both of which use CLIA-compliant laboratories^77^). We then address incidental findings initially found in research sequencing (which may or may not use CLIA-compliant laboratories) and communicated to the research participant or the participant's clinician for clinical evaluation. Finally, we address patients' right to access completed laboratory reports and the contents of the “designated record set” (DRS) in HIPAA-covered entities, as those reports and the DRS may contain incidental findings.

In clinical care, liability can result from a failure to analyze and communicate genomic incidental findings, from incorrect analysis of incidental findings (including a false positive), or from miscommunication of incidental findings. Liability could also result from failure to properly manage incidental findings ascertained in the research context and then communicated to clinicians because of their potential health implications.^78^

Clayton and colleagues published a 2013 analysis of legal cases to date in which clinicians faced assertions of liability for mishandling of incidental findings, focusing on cases involving imaging/radiology since no cases involving genetics had been reported at the time.^79^ They found that clinicians could face liability for a range of behaviors, if those behaviors breached the applicable standard of care. The behaviors included failure to recognize an incidental finding or appreciate its clinical significance, failure to notify the patient and/or the patient's clinician, and failure to refer the patient to a clinician with the needed expertise. In each of these cases, the patient would need to show that the failure caused harm — for example, by depriving the patient of a chance to seek care that could have averted harm or improved the patient's status. These authors, however, were careful to identify a number of reasons why liability should be less common in genetics.

In radiology, professional societies have formulated guidelines to assist radiologists and other clinicians in reading scans of various types and determining what incidental findings they should recognize, analyze, and report.^80^ There is less guidance and more controversy in clinical genomics. In 2013, an ACMG committee published the recommendation that whenever clinical sequencing was undertaken for any indication, the laboratory should routinely analyze 56 additional pathogenic genes to report these to the patient's clinician to avert harm.^81^ This recommendation was widely debated; objections included that this represented opportunistic screening without adequate evidence of likely net benefit, the initial recommendation failed to allow patients to consent to sequencing but to refuse ascertainment of these extra findings, and the recommendation applied to children even when the genes in question were irrelevant to the child's health before the age of majority because they involved adult-onset conditions.^82^ ACMG modified the recommendations to allow patients to opt-out of ascertainment of the extra findings and to enlarge the list from 56 to 59.^83^

The radiology analogy suggests that clinicians are obligated to recognize and report incidental findings when this is likely to avert or minimize harm to the patient or improve future health. However, the scope of genomic analysis that laboratories and clinicians are obligated to undertake remains unclear. If the lab discovers a pathogenic incidental finding within the scope of genomic analysis that was ordered, and reporting that finding is likely to avert or reduce harm, the lab is probably obligated to report that. However, the question remains how much further the lab should look in search of incidental findings. As an ethics matter, many scholars have argued against a “duty to hunt” for incidental findings, though much of that literature addresses the research context instead of the clinical one.^84^ The ACMG recommendations attempt to set up a process of offering limited extra analysis of a predefined set of incidental findings that a patient may accept or decline.

Once the laboratory has reported an incidental finding with health implications for the patient, the clinician is obligated to offer disclosure of that finding to the patient and obligated to manage the finding in keeping with the standard of care (either directly or through appropriate referral). Patients may decline to receive the finding, exercising what the ethics literature calls the “right not to know.”^85^ However, there is controversy about the scope of that right,^86^ and a clinician whose patient declines to know about a pathogenic finding that warrants clinical intervention or heightened surveillance faces a difficult situation that may warrant seeking ethics advice and legal counsel.

In the context of public health screening — including newborn screening and other forms of genetic screening on a population, subpopulation, or opportunistic screening basis — consideration of using larger panels, as well as exome and genome sequencing,^87^ raises the potential to identify incidental findings. Because public health screening is conducted using CLIA-compliant laboratories, communication of incidental findings to the clinician will trigger the same obligations that are triggered when clinically ascertained incidental findings are identified.

In the research context, an incidental finding may be defined as “a finding concerning an individual research participant that has potential health…importance and is discovered in the course of conducting research, but is beyond the aims of the study.”^88^ Once an incidental finding from research is communicated to a clinician because of its potential health implications, the clinician will need to seek CLIA confirmation of the finding (if it was originally ascertained in a non-CLIA laboratory) before clinical use of that finding. The clinician will then need to assess the health implications of the finding in the context of clinical evaluation.^89^

The question of whether researchers have duties to analyze and offer to the patient (or patient's clinician) incidental findings discovered in research is an evolving one. An extensive literature argues that researchers should offer back to research participants those findings that are pathogenic and clinically actionable, and that researchers may in their discretion offer back a larger set of findings.^90^ Milstein has argued that failure to offer back incidental findings that are pathogenic and clinically actionable may be malpractice.^91^ Wolf and colleagues have suggested that federal research regulations (the Common Rule and FDA equivalent) offer support for researcher duties to alert prospective participants to the possibility of discovering incidental findings and the researchers' plan for managing and disclosing them.^92^ In addition, the authors argue that the regulations support a duty to disclose, if such disclosure “may relate to the subject's willingness to continue participation” in the trial.^93^ A court may find negligence if the court finds that the researcher-participant relationship creates duties that run from the researcher to participant and that those duties may have been breached causing harm.^94^ When the researcher is also the participant's clinician, the court may find medical malpractice.^95^

Some authors have cautioned that expecting researchers to analyze and offer disclosure of incidental findings invites participants to confuse research for clinical care (in what is sometimes called the “therapeutic misconception”) and risks depletion of research budgets for a clinical activity.^96^ However, there appears to be wide agreement at this point that researchers should offer back to research participants at least those findings that are pathogenic and clinically actionable.^97^

When researchers ascertain incidental or secondary findings with potential health implications, they may seek to communicate those findings to the research participant for evaluation with the participant's clinician and may communicate them directly to the clinician. If those findings were ascertained or confirmed in a CLIA-compliant laboratory, the findings may be communicated and directly used in clinical care. If the findings were discovered in a non-CLIA certified laboratory, there is some disagreement over whether the findings can be communicated to the participant and/or clinician for the purpose of seeking clinical confirmation and evaluation.^98^

Ordering appropriate CLIA confirmation (if needed), interpreting the test results in the context of patient evaluation, and recommending next steps may generate liability if the clinician fails to meet the applicable standard of care. In addition, the clinician should appreciate the limits of their expertise and seek consultation with a medical geneticist, genetic counselor, or other clinician when needed.

Note that some genomic research is intrinsically translational, mixing research and clinical care.^99^ Such research often involves patients affected with illness, genomic sequencing to produce findings that may be relevant to care, inclusion of those findings in the medical record, and treatment decisions based on those findings.^100^ In such translational research, courts may find that there is a clinical duty to analyze and offer incidental findings to the patient-participants.

Finally, patients, individuals subject to public health screening, and research participants may access incidental findings by asserting their rights under federal law to access completed laboratory reports^101^ and their right of access to the contents of the DRS in HIPAA-covered entities.^102^ State law may also create rights of access. Once patients bring these incidental findings to the attention of their clinical caregiver, the clinician will need to deal with those findings responsibly, as outlined above.

##### B. recommendations

Recommendations for Health Care Actors or Institutions:

- Clinicians ordering genomic analyses including sequencing should be aware of key recommendations in the literature and from professional societies on analysis and return of incidental or secondary findings. Legal counsel should additionally be aware of the relevant law and legal analyses, in order to provide advice when needed.
- Clinicians should work with their genomics laboratory to clarify the scope of prospective analyses and how analysis and reporting of incidental or secondary findings will proceed. They should clarify how results will be reported, using what format, and with what entry in the EHR. They should also clarify how patients' requests for completed laboratory reports will be handled, and how patients will be able to access their data upon request.
- Genomics laboratories and institutions should formulate written policy on the scope of their analysis, including how they will manage incidental findings ascertained in clinical, public health, and research genomic analyses. Laboratories and institutions should have in place a system and operating procedures for ascertaining, recording, and communicating incidental findings. Laboratories and institutions should also clarify how patients' requests for their own data and completed laboratory reports will be handled, and whether patients will be given raw data or data files and in what format. Laboratory and institutional policy and practices on return of incidental findings and data should be publicly posted and communicated to patients.
- Clinicians ordering genomic analyses should be clear on what incidental or secondary findings the laboratory may discover and the patient's option to decline ascertainment and notification of such findings.
- When patients are offered clinical genomic analyses, they should be informed of what incidental or secondary findings may be discovered and allowed to opt-out.
- When a clinician is notified by patients or researchers of incidental findings discovered in research, the clinician should clarify whether the findings were ascertained in a CLIA-compliant laboratory. If they were not so ascertained, then the clinician should order confirmation in a CLIA-compliant laboratory before use in diagnosis, treatment, prevention, or health assessment, in keeping with the CLIA statute and regulations. The clinician will need to assess the clinical importance of confirmed findings in the context of performing a clinical evaluation of the patient.
- Researchers using genomic analyses should specify how they will manage incidental or secondary findings. Their protocol should specify what results (if any) will be offered, to whom, and how. Researchers should clarify whether results offered will originate from a CLIA-compliant laboratory, or will be confirmed in such a laboratory before being offered to the research participant, or will be offered for clinical confirmation and then evaluation.
- Clinicians, public health authorities, and researchers should specify their approach to return of results and incidental or secondary findings when the research participant is a minor, an adult without decisional capacity, or is deceased.
- Researchers should address how they plan to handle requests for the participant's raw data or data files.

Recommendations for Legal Development and Change:

- The DHHS Office of Civil Rights (OCR) should enforce patients' and research participants' existing rights of access to information in the DRS.
- Federal authorities (OCR and the Centers for Medicare & Medicaid Services (CMS)) should enforce patients' existing right of direct access to completed laboratory reports.
- Given that research may generate incidental findings for clinical evaluation and that some research analyses are conducted in non-CLIA laboratories, CMS should withdraw its 2014 pdf purporting to bar return of individual-specific results from such laboratories.^103^ When the results do not originate in a CLIA-certified (or CLIA-exempt) laboratory, CMS should acknowledge that the research team may confirm the result in such a laboratory or may communicate the result to the participant and/or clinician with clear warnings that the result is not being returned for diagnosis, treatment, or health assessment, and should be confirmed in a CLIA-certified (or CLIA-exempt) laboratory before clinical use.

#### 9. Failure to Update and Recontact

##### A. analysis

Genomic medicine will impose new opportunities and pressures for health care providers to recontact patients to provide updated interpretations of genomic information. Health care providers have always had a legal and ethical duty to try to recontact previous patients when they discover, or should have discovered, that their initial diagnosis or treatment recommendations were erroneous when originally provided.^104^ In the context of genomics, the question of whether the initial diagnosis, interpretation, or recommendation was erroneous will not be as clear-cut as in some other areas of medical malpractice. Given that a particular variant could be categorized as ranging from benign to pathogenic, and different databases may reflect divergent expert interpretations and classifications of the same result,^105^ it will not always be clear, even in retrospect, whether the initial decision of the physician to classify a variant or recommend a treatment regime based on that classification was erroneous at the time.

However, when the provider's actions were not erroneous at the time the advice was delivered, courts have recognized a duty to recontact patients in only two limited circumstances.^106^ The first is when a physician administers a procedure or treatment that requires an established follow-up procedure or test, and if such follow-up is not provided, the physician could be liable for “abandonment.”^107^ The second situation arises when a patient is being treated with a drug or device prescribed by the physician, and the physician learns of new risks associated with that treatment, such as a new warning issued by the FDA.^108^ The theory behind these two limited duties is that the patient is still undergoing treatment that the physician initiated.

Other than those limited situations, courts have not recognized a general duty for a health care provider to affirmatively recontact their patients with new information.^109^ Genomic sequencing presents new questions about whether a physician and testing lab can and should update results and recontact patients. Genome sequencing will initially identify many VUSs, but their clinical significance will change over time. This then raises the questions — which professional societies have begun to address — of whether there is a duty for testing labs or physicians to determine whether a variant's reinterpretation applies to previous patients, and if so, if the new information is clinically relevant, whether the testing lab and/or physician has any ethical or legal duty to try to recontact the patient with that updated information.^110^

A recent survey of the literature on a physician's legal duties to recontact a former patient to provide later findings identified a number of articles discussing a potential duty to recontact, such that the failure to do so could result in liability to former patients under a theory of negligence.^111^ Approximately one half of the articles reached no clear conclusion regarding whether there is a duty to recontact former patients to provide updated genomics findings; of 28 that drew a conclusion, “6 concluded that a duty to recon-tact does apply and 22 concluded that it does not.”^112^ Accordingly, scholars have concluded that “there is no generally held legal basis for recontacting in clinical genetics, although it is often considered desirable by both [health care providers] and patients.”^113^ However, no court has yet ruled on this potential legal duty in the context of updated genomic interpretations.^114^ A number of factors are relevant to any potential future ethical or legal duty to update genetic interpretations and recontact patients.

Imposing a responsibility on physicians to identify when new information changes the genetic risk for former patients is problematic, as most physicians will lack sufficient knowledge and confidence to identify and address the need for an update.^115^ Any potential future duty of physicians to update persons who receive genomic tests would likely vary depending on the nature, and inherent expectations, of the physician-patient relationship. The potential that the physician-patient relationship could give rise to a recognized duty to recontact or update patients could vary in at least three sets of circumstances, depending upon the relationship of the genomic results to the area of treatment.

First, a physician could order a genomic test that reveals a variant that is later determined to be associated with a disease risk unrelated to the physician's area of practice, and therefore outside the area of care that the patient received from the physician. In such a case, there would be a relatively lower risk that a court would impose liability on the physician for not recontacting and updating the former patient.

Second, the physician could order genomic sequencing in the course of providing continuing care to the patient. If the test identifies a genomic variant associated with a disease risk related to ongoing care, a court would be much more likely to determine that the physician has a duty to recontact and update the patient. However, a court might be less willing to impose liability on a physician in the context of offering ongoing treatment if the new genomic variant were in a field unrelated to the physician's area of practice and if the physician were unaware of the relevance of the new variant to the patient.

Third, there is the possibility of an intermediate scenario, in which the patient sees a physician for a discrete episode of care (*e.g.*, cardiac surgery and follow-up) after which the physician's relationship with the patient ends. If a genomic test were ordered in the course of the treatment relationship and then a variant identified by the genomic test were later determined to be associated with a disease risk related to the problem for which the patient sought care, it is unlikely that the physician would have a duty to recontact the patient and update regarding the risk; the physician has no ongoing relationship with the patient, and one episode of care cannot impose a lifetime, continuing duty on a provider. Indeed, some scholars have suggested that any duty to contact would be “unlikely to extend in perpetuity or to require more than reasonable effort.”^116^

In contrast to physicians, testing labs generally do have the requisite expertise to recognize and conduct reinterpretations of previous VUSs. Indeed, some labs are now periodically rechecking their previous interpretations and sending updated interpretations that are clinically relevant to the physician who ordered the genetic testing.^117^ However, there are no laboratory guidelines, court decisions, or other statements of standard of care that impose a duty on testing labs to periodically reinterpret genetic test results. Moreover, test labs generally do not have the immediate relationship or the proper training to report new genetic interpretations directly to patients.

If a responsibility to update genomic test results does emerge, it would likely put the onus on labs to identify the reinterpretation and then to send it to the ordering physician for communication to the patient. Yet, creating such a duty would involve significant practical and implementation issues.^118^ How long would the hypothetical duty to recontact extend into the future — for a limited time period or in perpetuity? Perhaps it would extend as long as there is a physician-patient relationship between the provider and patient at issue, but it is not always clear when such a relationship ceases. Is it when the doctor last sees the patient, or has not seen the patient for some length of time, or when the patient has changed their care to another physician? The end date of a physician-patient relationship is often not clear, and may depend on whether it was a one-time appointment with a specialist, a repeated series of appointments with a primary care physician, or perhaps a comprehensive care arrangement such as with an accountable care organization or institutional provider like Kaiser. In the past, a clear termination date for the physician-patient relationship may not have been legally significant in most cases, but given the looming possibility of an ongoing duty to update interpretations of genetic test results, it may behoove providers to contractually specify a more precise termination date for their care.^119^

In the future, information technology and patient apps may help make the reinterpretation tasks more routine and patient-focused. It is likely that computer programs and apps will be developed that automatically notify patients when clinically significant new information becomes available that affects the interpretation of their genetic results.

Many other practical difficulties confound a possible ongoing duty to update. Who would pay for the additional efforts by the lab and physician? What if the patient has moved and is not easily located? What if the patient did not want to know the updated results? What if the patient has died in the interim? These and other practical difficulties in implementing a possible duty to update should be resolved before any such duty is imposed.

In the future, information technology and patient apps may help make the reinterpretation tasks more routine and patient-focused. It is likely that computer programs and apps will be developed that automatically notify patients when clinically significant new information becomes available that affects the interpretation of their genetic results. Indeed, a program called “GenomeConnect: The ClinGen Patient Registry” has already been launched that among other things alerts patients to significant updates in the interpretation of their genetic test results.^120^

##### B. recommendations

Recommendations for Health Care Actors or Institutions:

- If a provider discovers that the previous information communicated to the patient was erroneous at the time of the communication, the provider should notify the patient of the corrected information as soon as possible.
- Health care providers who are responsible for ordering or interpreting genetic tests for a patient should expressly inform the patient that some of the results may have a different or changing interpretation in the future.
- Some testing labs are periodically updating their interpretations of previous patient test results in light of constantly evolving information. If during such a review, the lab determines that the interpretation of a patient's genetic information has changed to now provide an actionable response to a significant risk, the lab should make reasonable efforts to communicate that revised finding to the ordering physician.
- If a physician is notified by a testing laboratory that a previous interpretation of a patient's genetic variant has changed in such a manner that may materially affect the patient's health, the physician should make reasonable efforts to pass on the updated interpretation to the patient. In many or even most cases, this may not be feasible, due to patient loss to follow up and limited provider resources.
- A physician who is treating a patient for a condition and who refers the patient for a genetic consultation that provides no clinically relevant information should consider re-referring the patient for an updated genetic consultation every few years if clinically indicated (*e.g.*, condition has not been resolved and is not being successfully treated and physician has reason to suspect there may be a previously undetected genetic underpinning).
- Any duty of a physician to recontact and update a patient regarding newly identified genomic testing results should depend on various factors including: (1) the correspondence, if any, between the physician's area of expertise and the genomic result, (2) whether the patient is under the physicians' current and continuing care, and (3) the period of time that has elapsed since the physician's care.
- In the future, software programs and apps may become available to automatically notify patients and their treating physician that a clinically relevant updated interpretation of their genomic data is available.

Recommendations for Legal Development and Change:

- Courts and legislatures have to date established no general legal duty for a test lab or health care provider to continuously update previous interpretations of a patient's genetic variant(s).
- A provider who is actively treating a patient for a specific condition, and that patient has been genetically tested in the past, should be held legally responsible for taking reasonable steps to communicate updated clinically-relevant genetic information to the patient if the updated information has been communicated to the physician and that information is within the physician's scope of expertise, or would otherwise be within the standard of care for the physician to be aware of the updated information.
- A health care provider who is provided with a materially updated interpretation of a patient's genetic information from a test lab should try to make reasonable efforts to pass that information on to the patient. Although no legal duty currently exists for a provider to communicate those updated test results to their patient, providers should be aware that courts may impose such a duty in the future, perhaps retroactively. However, legal systems should understand that this recontact is in many, even most, cases not feasible, given patient loss to follow up and limited provider resources, and thus should condition any such duty on a reasonableness determination.
- Courts and legislatures to date have imposed no general duty on testing labs to regularly update the results from previous patients or to alert ordering physicians of clinically relevant updates. However, given that some labs have started to do such updates as a regular part of their business, they may be establishing a new standard of care, especially in jurisdictions where test labs are considered to be health care providers that are subject to a standard of care based on local or national custom.
- Professional societies should continue to refine their guidance and provide recommendations on what is a reasonable effort that providers and test labs should undertake to update patient information with relevant new findings. Relevant factors should include the nature of the patient-provider interaction, the duration since the last patient appointment, whether the patient has provided the provider with their most recent address and contact information, the clinical significance of the new findings, and how often any update required should occur.

#### 10. Failure to Warn Family Members

##### A. analysis

Health care providers have been concerned for many years about potential liability relating to failing to warn family members of patients about their genetic risks.^121^ Genetic information differs from most other medical data in that it is directly relevant to family members who may share the patient's genetic variants that confer a disease risk. Courts have held that a health care provider has a legal duty to warn relatives of a patient of genetic risks that become apparent through treatment of the patient by instructing the patient to warn their immediate family members of their potential genetic risk.^122^ However, with some limited exceptions discussed below, courts have generally not held that providers have a duty to communicate genetic information to a patient's relatives directly, and such disclosure without the patient's consent is generally prohibited by the Health Information Portability and Accountability Act (HIPAA). Some medical association guidelines such as those from the American Society of Human Genetics (ASHG) and National Society of Genetic Counselors (NSGC) suggest that a health care provider may (but it is not required to) directly disclose genetic risk information to a patient's relatives in the absence of the patient's consent in rare, exceptional circumstances.^123^ Some experts contend, however, that any such narrow discretionary right to disclose genetic information to a patient's relatives was foreclosed by the 2003 privacy rule implementing HIPAA (discussed further below).^124^

This duty to warn the patient to disclose family risks may be more difficult and complex to discharge in the era of genomics than genetics. With genetics, usually a mutation in a single gene is at issue, and therefore it is relatively straightforward for a provider to discern and communicate to the patient the inheritance pattern of that mutation (*e.g.*, dominant vs. recessive, autosomal vs. sex-linked). The potential risks to immediate family members can then be conveyed to the patient with a recommendation that such information be passed on to blood relatives. With genomic test results, including test results of a large number of gene variants with varying degrees of significance, the task of determining what information (if any) should be communicated to the patient's relatives is much more difficult. For example, some of the mutations in a patient's genome may have been inherited, while others may be *de novo*, especially with mutations such as copy number variants (CNVs). Even for variants that appear to have been inherited, there is a lack of clarity on how much clinical significance and certainty about a variant is necessary to trigger a duty by the provider to counsel the patient to communicate the risk information to relatives. Even if a physician's duty is discharged by recommending information for the patient to tell his or her direct relatives, the shift from genetics to genomics will make this legal duty more complex and potentially subject to retrospective criticism in any litigation.

At the same time, the shift from single-gene genetics to genomics in health care may create more cases where there may be a duty to disclose to relatives for a couple of reasons. First, as a purely quantitative matter, as more people get their genes tested, there will be more cases where communication of those results to relatives will be clinically important. Second, to the extent that genomics progresses to various types of population-level screening, this too will create more opportunities and legal risks relating to disclosing information to relatives. For example, if sequencing of newborns becomes more prevalent, this will create enormous additional family disclosure issues. An example of a genomic test creating a duty to relatives is *Polaski v. Whitson*, where the court ruled that a physician treating a patient for heart disease may have legally harmed the patient's son by failing to order a gene panel test for the father for hypertrophic cardiomyopathy, which might have then informed the son's disease risk.^125^

The increased number of potential cases where genetic information may be relevant to a patient's relatives is offset at least partially by the fact that as more people undergo genome testing, they will no longer need to receive their relatives' results to learn of risk, since they will have the results from their own genome. However, the long-standing fear of many physicians is that the duty to disclose to relatives could be imposed directly on the physician, such that the physician will be tasked with informing the relatives directly if the patient is unwilling or incapable of doing this. This fear was catalyzed by the 1996 decision in *Safer v. Pack*,^126^ where a New Jersey court suggested that a doctor may have such a duty in at least some circumstances. The New Jersey legislature subsequently overturned the decision by enacting a genetic privacy statute that required patient consent for disclosure,^127^ and no subsequent court case in the United States has held that a physician has a legal duty to inform relatives of their genetic risk over the patient's objections (or even without the patient's permission).^128^

To obligate physicians to warn relatives directly would be problematic on both practical and legal grounds. First of all, disclosing over a patient's objections violates the clinician's millennia-old obligation to protect confidentiality. From a practical perspective, if a patient is uncooperative and unwilling to notify their own relatives, it will often be difficult for a physician to identify and locate at-risk relatives, who may be scattered in faraway places. The costs and burdens of contacting and communicating genetic risks to such relatives would likely not be reimbursed and could be quite substantial. Moreover, the relatives who receive the genetic warnings may not want to be informed of their genetic risks, having not consented to any communication, or may even be minors.

Legally, disclosing a patient's health information to relatives without the patient's approval could violate the HIPAA privacy rule.^129^ There are HIPAA exceptions to the consent usually required for the disclosure of medical information by a health care provider or institution that could theoretically apply to the unconsented disclosure of genetic risk information to the patient's relatives, including disclosures “to avert a serious threat to health or safety” or where “required by law.” These exceptions have primarily been applied to imminent and serious risks from contagious diseases or mental health problems. While there has been some debate in the academic literature about whether either of these provisions could apply to familial disclosure of genetic information, the nature of harm from non-disclosure of genetic information is unlikely to rise to the level of “serious threat” of harm and is not included with the regulatory explanation of the “required by law” exception.^130^ Furthermore, the 2013 amendments to the HIPAA rules do not expressly extend these HIPAA exceptions to cover sharing genetic results with a patient's relatives without the patient's consent.

HIPAA does allow a physician to send results about a patient's genetic risks to another provider treating a relative of the first patient, provided the patient has not forbidden such sharing.^131^ This allows sharing genetic results within families as more and more providers consider genetic data in their clinical care of patients. However, if the patient requests that information not be shared through this mechanism, *and* the physician agrees with that restriction, then the physician may not share genetic data with a physician treating a relative.^132^ Some other exceptions may apply in certain situations. Researchers and other actors outside the clinical context that work in institutions that are not “covered entities” under HIPAA are not precluded by HIPAA from sharing genetic test results with relatives in appropriate circumstances.^133^ Moreover, some states assign the rights to medical information (including genetic data) to relatives of a deceased patient.^134^

Finally, because HIPAA allows a personal representative of the patient to gain access to a patient's medical information, some relatives will gain access to patient genetic data through that mechanism.^135^

However, as a general matter, a physician has no duty to disclose patient genetic risk information to the patient's relatives, but may risk violating HIPAA if the physician attempts to disclose such information without the patient's consent (and the patient is still alive^136^).

##### B. recommendations

Recommendations for Health Care Actors or Institutions:

- Clinicians ordering genomic analyses including sequencing should be aware of key recommendations in the literature and from professional societies on sharing results and data or data files with family member(s) or their physician(s), including after the patient's loss of decisional capacity or death. Legal counsel should additionally be aware of the relevant law and legal analyses, in order to provide advice when needed.
- Genomic data and interpreted results qualify as an individual's protected health information (PHI) and should generally be protected as private and confidential information. Researchers and clinicians should encourage individuals to share their results with family member(s) when those results have implications for those family member(s), and should offer support for that process as needed. It may be particularly helpful to provide the patient with an informational document to share with family members.
- Clinicians should specify their approach to disclosure of results to family members when the patient is a minor, an adult without decisional capacity, or is deceased. Clinicians should consider eliciting the patient's preferences on sharing results with family members, including after participant loss of decisional capacity or death.
- Clinicians should address how they plan to handle requests for patient results from the patient's family member(s), parent(s) or guardian, the patient's Legally Authorized Representative (LAR), or the patient's Personal Representative (under state and federal law); the LAR and/or the Personal Representative may be a family member.
- Clinicians and test labs should address how they plan to handle requests for the patient's raw data or data files from any of those listed above.
- There may be cases in which the patient refuses to share results that have a high likelihood of averting imminent harm if shared. In these instances, the clinician may have a privilege to warn the family member(s) at risk, although some legal experts argue that any such disclosure would violate HIPAA. Ethics and legal consultation is recommended before sharing.

Recommendations for Legal Development and Change:

- Provisions in HIPAA and state law allowing broader sharing of genomic PHI with family members should be scrutinized and amended if necessary to ensure that sharing of genomic PHI without authorization from the source individual is limited to reliable results of high value to the family member(s) in question and that the benefit to the family member(s) warrants the privacy risks to the source individual, including after death.
- Courts and other actors should clarify where necessary that under current law health care providers do not have a duty to disclose genetic test results to relatives of a patient if the patient does not consent to such disclosure.
- Some courts have held that a person has a cause of action and standing to bring a lawsuit against a clinician for negligent genetic testing of a family member, on the theory that the erroneous testing or reporting of the test results injured the relative bringing the lawsuit (*e.g.*, by depriving the plantiff of accurate genetic information about their relative that may have also pertained to them).^137^ Such lawsuits have the potential to significantly expand the duty and liability exposure of clinicians. If similar claims are recognized in the future, courts should clearly establish limits on any such duties to protect clinicians from overly-expansive liability exposure

#### 11. Errors and Failures Direct-To-Consumer (DTC) Testing

##### A. analysis

Direct-to-consumer (DTC) genetic testing is a relatively new arrangement for genetic testing that is likely to become even more common in the future as regulatory restrictions are relaxed and more consumers become interested in obtaining their own genomic information. In DTC genetic testing, a company sends a test kit directly to a consumer who purchases testing; the consumer provides a buccal swab, spit, or blood sample in a vial that is mailed back to the DTC company; then the DTC company after analyzing the sample provides the genetic test results directly to the consumer, usually through a password-protected web-site. The patient's personal physician is usually not involved in the ordering or initial receipt of the genetic test results, although the DTC company may have a physician on staff who technically “orders” the genetic testing to comply with regulatory requirements.^138^ However, many patients may bring their DTC genetic results to their personal physician for consultation and advice.

Providing genetic results through DTC companies raises many controversial policy and ethical concerns,^139^ and the FDA has been increasingly involved in regulation of DTC testing. Our focus here is on potential liability issues faced by a health care provider who is presented with or reviews a patient's DTC test results.^140^

DTC genetic testing can present liability risks for providers. For example, if a patient seeks to share with their physician DTC results that show disease susceptibility or PGx information about a certain pharmaceutical (*e.g.*, warfarin), and the physician knows of these results but fails to take them into account in providing medical advice, the physician may be faced with a medical malpractice claim, if that failure directly resulted in adverse effects on the patient. However, given that many DTC results, especially if self-reported, may not be accurate or reliable, any such physician duty to take such results into account must be tempered, and indeed, no cases have been reported in which physicians or other providers have been held liable for failing to consider DTC results in their care and treatment. If the DTC test results are potentially clinically significant, the best practice is for the patient's physician to order the test be validated in a CLIA-approved or CLIA-exempt laboratory, so that the results will be reliable. Nonetheless, a physician who ignores a patient's DTC results without getting the results validated, especially when brought to the provider's attention by the patient, may be at risk of liability.

Alternatively, a physician could be liable for over-relying on DTC genetic test results and recommending prophylactic surgery, pharmaceutical treatment, or some other unnecessary and possibly risky procedure without adequately validating the DTC genetic results.^141^ Recent studies have shown that DTC results are often erroneous,^142^ and even the DTC companies generally recommend that patients validate DTC results before relying on them for clinical decision-making. A physician who relies on DTC results without validating the results therefore also faces a liability risk. However, the physician may be put in a catch-22 situation if the patient's insurer will not pay for the clinical validation, and the patient cannot afford to pay for the validation test out-of-pocket. The physician then faces the dilemma of either relying on a potentially erroneous test result or alternatively ignoring a test result that may be valid. Either way, the patient may be harmed and the physician may be sued.

##### B. recommendations

Recommendations for Health Care Actors or Institutions:

- DTC genetic test results will usually include results from tests that are not clinically indicated. Such testing provides results that are more likely to be false positives and are not clinically useful compared to genetic testing in symptomatic or at-risk patients that is clinically indicated. Clinicians should be wary of relying on results from DTC tests which are not clinically indicated, and should require a confirmatory test before making clinical diagnoses or recommendations. Obtaining confirmatory testing could be problematic if, for example, the patient does not have insurance willing to pay for such confirma-tory testing.
- DTC genomics companies should ensure that they clearly explain the limitations of their tests and coverage, and in particular the potential for erroneous results and the potential for other untested variants to present risk to the patient.
- Providers delivering general care as well as more specialized care should be informed of the limitations and risks of DTC genetic testing, as well as any benefits in terms of costs and privacy, and be prepared to counsel their patients about such issues if and when patients inquire about such testing.
- Providers should not refuse to consider genetic information that a patient offers to share with their physician when there are reasonable grounds to believe that information may relate to a specific complaint or condition the patient is presenting. However, clinicians generally do not have the knowledge or resources to be the primary explainer of the DTC test results that all their patients receive, and should not be expected to review and understand all the relevant information that may be contained in those test results.
- A provider should not base clinical decisions solely on DTC test results. Rather, if the DTC test results indicate a potential problem or meaningful health indication, the provider should seek to have the test result replicated by a CLIA-compliant test laboratory.

It is important to emphasize that liability is only one in a set of tools that should be used together in promoting genomic medicine that is high quality, effective, cost-effective, ethical, and accountable. Education and training, clinical guidelines, practice support tools, corporate management and accountability, open sharing of data and methods, and individual and institutional ethics are all vital tools as well. Liability can be an important component of that toolbox, but if applied too broadly can impede the uptake of genomic medicine, and if applied too sparsely and inconsistently, will not play the role it should in protecting patients and promoting good care and the successful implementation of genomic medicine.

Recommendations for Legal Development and Change:

- When a provider fails to take into account specific actionable genetic test results (from any reliable source) that the patient brings to the provider's attention relating to a health condition or risk that the provider is treating, and the failure to consider that genetic information causes harm to the patient, the provider may be held liable for medical malpractice. However, providers should not be assigned the legal duty to be aware of and to take action on all the information in patient DTC genetic test results, in particular genetic test results from tests that are not clinically indicated, except for the narrow exception described above.
- A provider who recommends clinical action based on the results of a DTC genetic test, without first verifying the validity of those DTC test results, may be held liable for harm that results to the patient from the reliance on inaccurate and unverified DTC test results.

### Conclusion

Just as the transition from genetics to genomics will both transform and complicate clinical care, so too will it transform and complicate liability for health care providers. In this article, we have projected some of the general themes and trends. We then address eleven specific liability topics that will be important in the era of genomics medicine. Some of these topics merely represent an expansion of the importance and frequency of legal issues that have been with us since the onset of genetic testing, whereas others presentative novel legal issues that are unique to genomics. In both cases, these liability issues will challenge and exert pressure on actors in both the medical and legal professions to respond in appropriate and innovative ways. We have provided our recommendations on how both the health care and legal systems should respond, hoping to stimulate debate on these tough questions, recognizing that our recommendations may be just the first shot rather than the final word on how these difficult but important liability issues are resolved.

Predicting how the various liability risks relating to clinical genomics will manifest in actual cases is difficult given that the outcome of such claims will often depend on the specific facts, parties, attorneys, experts, judge, and jury. The early court decisions will have a big influence on the future feasibility and frequency of similar claims, as both medical and legal actors will rely on those initial decisions. The precise evolution of these liability claims is unpredictable, but litigation in this realm will likely follow the historical trend that new medical technologies breed new waves of malpractice liability lawsuits.^143^

It is important to emphasize that liability is only one in a set of tools that should be used together in promoting genomic medicine that is high quality, effective, cost-effective, ethical, and accountable. Education and training, clinical guidelines, practice support tools, corporate management and accountability, open sharing of data and methods, and individual and institutional ethics are all vital tools as well. Liability can be an important component of that toolbox, but if applied too broadly can impede the uptake of genomic medicine, and if applied too sparsely and inconsistently, will not play the role it should in protecting patients and promoting good care and the successful implementation of genomic medicine.
